# Muyocopronones A and B: azaphilones from the endophytic fungus *Muyocopron laterale*

**DOI:** 10.3762/bjoc.16.177

**Published:** 2020-08-28

**Authors:** Ken-ichi Nakashima, Junko Tomida, Tomoe Tsuboi, Yoshiaki Kawamura, Makoto Inoue

**Affiliations:** 1 Laboratory of Medicinal Resources, School of Pharmacy, Aichi Gakuin University, 1-100 Kusumoto-cho, Chikusa-ku, Nagoya, Aichi, Japan; 2 Department of Microbiology, School of Pharmacy, Aichi Gakuin University, 1-100 Kusumoto-cho, Chikusa-ku, Nagoya, Aichi, Japan

**Keywords:** azaphilones, endophytic fungus, modified Mosher’s method, *Muyocopron laterale*, polyketides

## Abstract

Two new azaphilones, namely muyocopronones A (**1**) and B (**2**), were isolated from the cultures of an endophytic fungus *Muyocopron laterale* ECN279. Their structures were elucidated by extensive spectroscopic analysis. Their absolute configurations were determined using the modified Mosher’s method and through comparisons of experimental and calculated electronic circular dichroism data. In addition, muyocopronone B (**2**) was found to exhibit a weak antibacterial activity against some Gram-positive bacteria.

## Introduction

Azaphilones, which are a class of fungal polyketides with diverse structures, have received growing attention due to their various biological activities, such as the inhibition of some protein–protein interactions [1–2], tau aggression [3], and heat shock protein 90 [4], in addition to their antimicrobial, cytotoxic, anticancer, and anti-inflammatory effects [5]. To date, over 400 azaphilones have been reported from various fungal strains, the majority of which have been produced by ascomycetes belonging to the classes Eutiomycetes (e.g., *Monascus*, *Penicillium*, and *Aspergillus* spp.) and Sordariomycetes (e.g., *Chaetomium*, *Hypoxylon*, and *Diaporthe* spp.) [5]. In addition, some of the azaphilones have been also found in the Dothideomycetes class such as *Pithomyces* [6], *Cochliobolus* [7–8], and *Leptosphaeria* spp. [9]. Interestingly, the taxon of the genus *Muyocopron* (Dothideomycetes), to which the strain examined in this study belongs, has been reevaluated based on phylogenetic and morphological analyses, and as a result, it has been proposed that some species previously included in *Mycoleptodiscus* spp. (such as *Muyocopron sahnii*, syn. *Mycoleptodiscus indicus*) are members of the *Muyocopron* genus [10]. Therefore, three azaphilone dimers [11] and a triterpenoid [12] previously isolated from *Mycoleptodiscus indicus* are now considered to be metabolites of *Muyocopron* strains. However, little information exists regarding the fungal metabolites produced by *Muyocopron* spp. Thus, as part of our research into the natural products produced by plant-associated fungi [13–15], we isolated two new azaphilones from the cultures of an endophytic fungus, namely *Muyocopron laterale* ECN279. Herein, the isolation, structural elucidation, and antimicrobial activity evaluation are described.

## Results and Discussion

**Figure 1:**
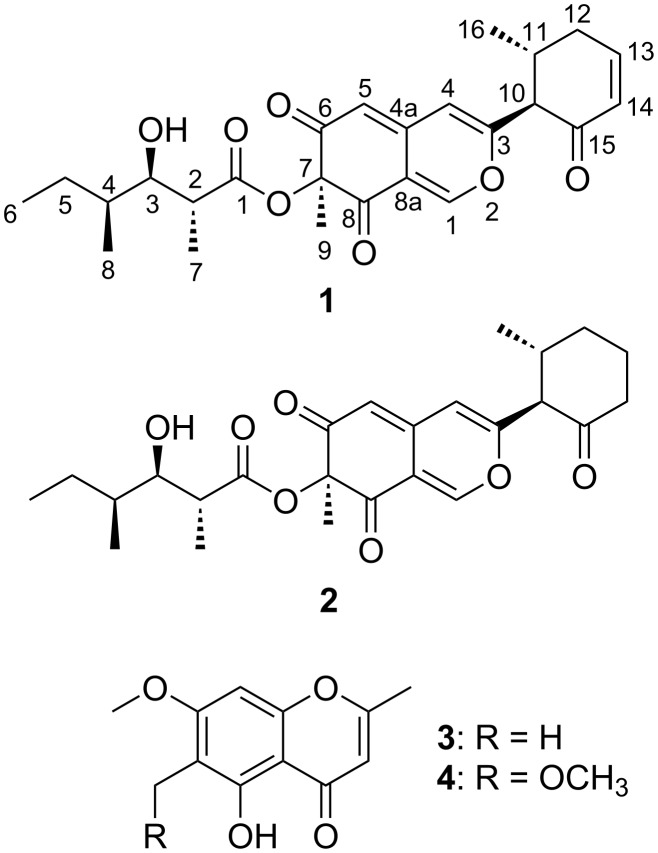
Structures of compounds **1**–**4**.

*Muyocopron laterale* ECN279 was isolated from a healthy leaf of *Canavalia lineata* and identified by sequencing the D1/D2 26S rRNA gene and the internal transcript spacers (ITS) of ribosomal DNA [16]. In a similar manner as described in our previous paper [14], the whole mycelia, which were cultured on 2% malt extract agar, were extracted with methanol (MeOH) at room temperature and the extracted solution was evaporated to obtain the crude extract. The crude extract was then partitioned between ethyl acetate and water, and muyocopronones A (**1**) and B (**2**) were isolated from the ethyl acetate layer together with eugenitin (**3**) [17], and 6-methoxymethyleugenin (**4**) [18]. The structures of compounds **3** and **4** were identified on the basis of their ^1^H and ^13^C NMR data (Figure 1).

**Figure 2:**
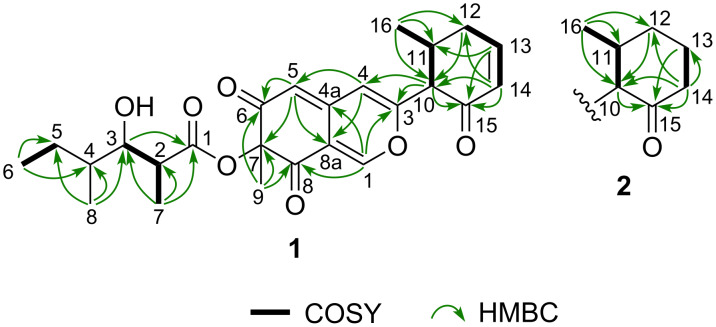
Key HMBC (green arrows) and COSY (bold) correlations in **1** and **2**.

Muyocopronone A (**1**) was isolated as yellow amorphous solid with a molecular formula of C_25_H_30_O_7_, as confirmed by a protonated molecular ion at *m*/*z* 443.2073 in the HRESIMS. The IR spectrum exhibited absorptions corresponding to the hydroxy (*ν*_max_ 3468 cm^−1^) and carbonyl (*ν*_max_ 1717, 1674, and 1633 cm^−1^) groups. In addition, in the ^1^H NMR spectrum (Table 1), signals were observed corresponding to five methyl groups [δ_H_ 0.90 (d, *J* = 6.4 Hz, 3H, H_3_-8′), 0.93 (t, *J* = 6.9 Hz, 3H, H_3_-6′), 1.07 (d, *J* = 6.4 Hz, 3H, H_3_-16), 1.18 (d, *J* = 6.9 Hz, 3H, H_3_-7′), and 1.60 (s, 3H, H_3_-9)] and five sp^2^ methine groups [δ_H_ 5.56 (d, *J* = 0.9 Hz, 1H, H-5), 6.14 (dd, *J* = 2.5, 10.1 Hz, 1H, H-14), 6.22 (s, 1H, H-4), 7.08 (ddd, *J* = 2.3, 5.5, 10.1 Hz, 1H, H-13), and 7.87 (d, *J* = 0.9 Hz, 1H, H-1)]. The ^13^C NMR and DEPT135 spectra (Table 1) showed 25 carbon resonances corresponding to five methyl groups, two sp^3^ methylene groups, five sp^3^, and five sp^2^ methine groups, in addition to one sp^3^ and seven sp^2^ non-protonated carbon atoms, including one ester carbonyl at δ_C_ 175.2 (C-1′) and three conjugated carbonyl carbons at δ_C_ 193.0, 193.3 (C-6 and C-8, interchangeable), and 194.4 (C-15). The presence of a 2,4-dimethyl-3-hydroxyhexanoate moiety was indicated by the DQF-COSY sequences of H-3′/H-2′/H_3_-7′ and H-5′/H_3_-6′, and the HMBC correlations of H_3_-6′/C-4′, C-5′, H_3_-7′/C-1′, C-2′, C-3′, and H_3_-8′/C-3′, C-4′, C-5′ (Figure 2). Furthermore, a 6-methyl-2-oxocyclohex-3-en-1-yl substituent was confirmed by the DQF-COSY sequences of H-10/H-11/H_3_-16 and H_2_-12/H-13/H-14, and the HMBC correlations of H_2_-12/C-10, H-13/C-11, C-15, H-14/C-10, C-12, and H_3_-16/C-10, C-11, C-12. The chemical shifts of the remaining three sp^2^ methine groups (H-1, H-4, and H-5), a singlet corresponding to the methyl group (H_3_-9), and six non-protonated carbon atoms (C-3, C-4a, C-7, and C-8a) including two conjugated carbonyl carbon atoms (C-6 and C-8), closely resembled the chemical shifts of the azaphilone skeleton observed in S-15183a and S-15183b [19]. The presence of an azaphilone skeleton was also corroborated from the HMBC correlations of H-1/C-3, C-4a, C-8, H-4/C-5, C-8a, H-5/C-7, C-8a, and H_3_-9/C-6, C-7, C-8. Furthermore, the 6-methyl-2-oxocyclohex-3-en-1-yl group was found to be located at the C-3 position of the azaphilone core, as observed by HMBC correlations of the methine proton signal at δ_H_ 3.09 (d, *J* = 12.8 Hz, 1H, H-10) with the C-3 and C-4 carbon signals. According to the molecular formula determined by HRESIMS, the 2,4-dimethyl-3-hydroxyhexanoate side chain was finally predicted to be connected to the remaining oxygenated carbon (C-7) of the azaphilone core.

**Table 1:** ^1^H and ^13^C NMR spectroscopic data for **1** and **2**^a^.

	muyocopronone A (**1**)		muyocopronone B (**2**)	
Position	δ_C_, type	δ_H_, mult. (*J* in Hz)	δ_C_, type	δ_H_, mult. (*J* in Hz)

1	154.2, CH	7.87, d (0.9)	154.2, CH	7.91, d (0.9)
3	157.5, C		157.7, C	
4	113.3, CH	6.22, s	112.5, CH	6.16, s
4a	115.1, C		114.9, C	
5	107.3, CH	5.56, d (0.9)	106.9, CH	5.53, d (0.9)
6	193.0^b^, C		193.2^b^, C	
7	84.7, C		84.6, C	
8	193.3^b^, C		192.9^b^, C	
8a	142.1, C		142.4, C	
9	22.1, CH_3_	1.60, s	22.0, CH_3_	1.59, s
10	59.4, CH	3.09, d (12.8)	62.4, CH	3.05, d (11.9)
11	32.7, CH	2.55, m	36.5, CH	2.20, m
12α	33.9, CH_2_	2.24, dddd (2.3, 2.5, 10.1, 18.8)	33.0, CH_2_	1.53^c^, m
12β		2.60, ddd (5.0, 5.5, 18.8)		2.02, m
13α	150.4, CH	7.08, ddd (2.3, 5.5, 10.1)	25.0, CH_2_	1.75, m
13β				2.12, m
14α	128.9, CH	6.14. dd (2.5, 10.1)	41.0, CH_2_	2.52, br d (14.1)
14β				2.36, ddd (6.2, 14.0, 14.1)
15	194.4, C		205.8, C	
16	19.7, CH_3_	1.07, d (6.4)	20.6, CH_3_	1.03, d (6.4)
1′	175.2, C		175.0, C	
2′	43.1, CH	2.79, dq (6.9, 8.2)	43.1, CH	2.79, dq (7.3, 8.2)
3′	76.1, CH	3.66, dd (3.2, 8.2)	76.0, CH	3.67, dd (3.2, 8.2)
4′	36.1, CH	1.53^c^, m	36.0, CH	1.53^c^, m
5′	26.7, CH_2_	1.36, m	26.6, CH_2_	1.36, m
		1.53^c^, m		1.53^c^, m
6′	11.7, CH_3_	0.93, t (6.9)	11.7, CH_3_	0.93, t (7.3)
7′	13.5, CH_3_	1.18, d (6.9)	13.4, CH_3_	1.18, d (7.3)
8′	12.2, CH_3_	0.90, d (6.4)	12.1, CH_3_	0.90, d (6.9)

^a1^H NMR spectra were measured at 400 MHz, and ^13^C NMR spectra were measured at 100 MHz. Overlapped signals were assigned based on the DQF-COSY, HSQC, and HMBC spectra. ^b^Interchangeable. ^c^Overlapping signals.

**Scheme 1:**
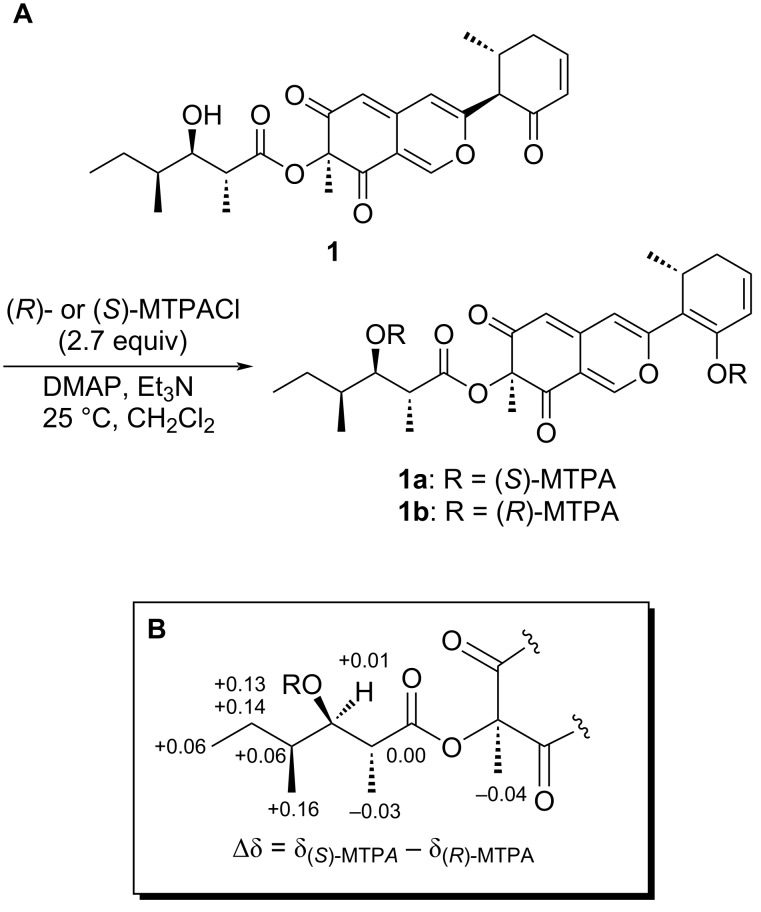
(A) Synthesis of MTPA diesters **1a** and **1b**. (B) Δδ Values for the (*S*)- and (*R*)-MTPA esters of **1**.

During their investigation into the structural determination of (−)-SCH 64874, Tokuyama et al. reported the synthesis and NMR characterization of the four possible diastereomers of the β-hydroxycarboxylic acid side chain [20]. Based on the obtained NMR data, the relative structure of the side chain of **1** was determined to adopt the (2′R*,3′R*,4′S*) configuration, as determined by the chemical shifts and the coupling constants between H-2′/H-3′ (*J* = 8.2 Hz) and H-3′/H-4′ (*J* = 3.2 Hz). To establish the absolute configuration of the side chain, we therefore employed the modified Mosher’s method [21]. Although we initially attempted the preparation of the α-methoxy-α-trifluoromethylphenylacetic acid (MTPA) esters using 1.2 equivalents of MTPA chloride, enolization and esterification at the C-15 position occurred preferentially over the C-3′ position. Therefore, 2.7 equivalents of the (*R*)- and (*S*)-MTPA chlorides were used to prepare the (*S*)- and (*R*)-MTPA diesters **1a** and **1b**, respectively (Scheme 1A). Based on the differences in chemical shifts between **1a** and **1b** (Δδ_(_*_S_*_)-MTPA−(_*_R_*_)-MTPA_), the absolute configuration of the side chain was established to be the (2′*R*,3′*R*,4′*S*) configuration (Scheme 1B).

**Figure 3:**
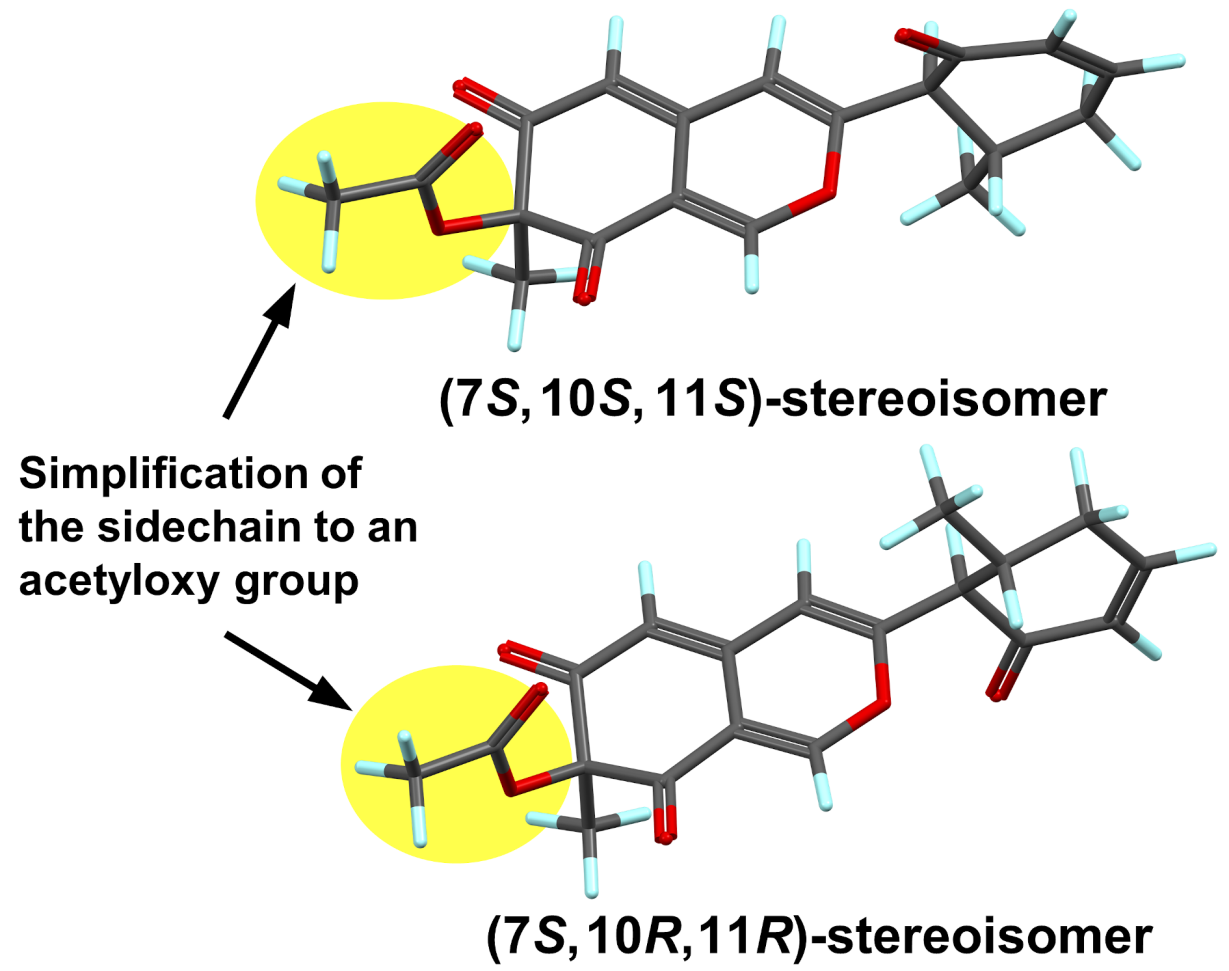
Simplified model structures for calculations of the ECD spectra of **1**.

**Figure 4:**
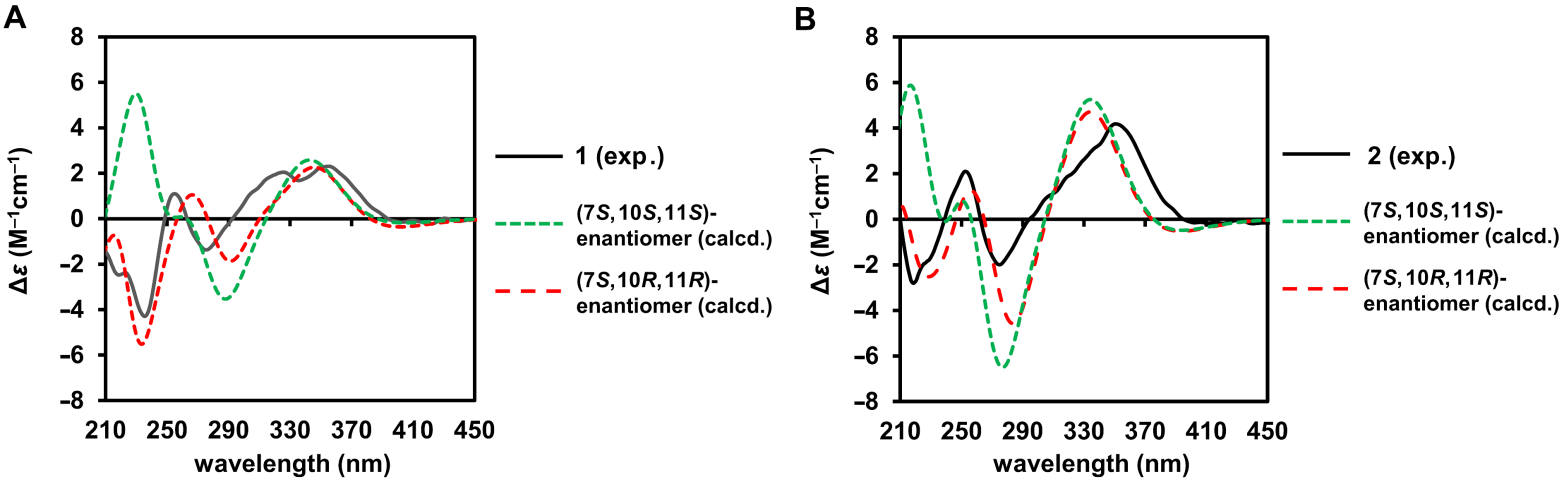
Comparison of the experimental ECD spectra (black solid line) of **1** (A) and **2** (B) with the Boltzmann-weighted spectra computed for the (7*S*,10*S*,11*S*)- and (7*S*,10*R*,11*R*)-stereoisomers (green and red dashed lines, respectively) of the simplified models.

The absolute configuration at the C-7 position of the azaphilones can be established by the positive and negative Cotton effects at the longest wavelength around 360 nm corresponding to 7*S* and 7*R* [22–24]. Therefore, positive Cotton effects at 325 and 355 nm in the electronic circular dichroism (ECD) spectrum of **1** indicated that the absolute configuration of the C-7 position was *S* (Figure 3). Furthermore, the coupling constant between H-10/H-11 (*J* = 12.8 Hz) indicated that H-10 and H-11 were in *trans*-configured, i.e., (10*R*,11*R*)- or (10*S*,11*S*)-configurations. However, the relative stereochemical relationship between C-7 and C-10/C-11 could not be determined by experimental data. Therefore, we conducted comparisons of the calculated and experimental ECD spectra to elucidate the absolute configuration. In the quantum-chemical calculations, the generation of an excessive number of conformers was avoided using a molecular model in which the β-hydroxycarboxylic acid side chain at the C-7 position was simplified to an acetyloxy group (Figure 4A) [25]. After conformational analysis, geometry optimization was performed for two possible stereoisomers with the (7*S*,10*R*,11*R*)- and (7*S*,10*S*,11*S*)-configurations using density functional theory (DFT) at the CAM-B3LYP/6-311+G(d,p) level of theory. In addition, the ECD spectra of the DFT-optimized conformers were calculated using time-dependent DFT (TDDFT) at the B3LYP/6-311+G(d,p) level of theory. The negative Cotton effect observed at 236 nm in the measured spectrum was in good agreement with that in the calculated ECD spectrum of the (7*S*,10*R*,11*R*)-stereoisomer (Figure 4A). Thus, the absolute configuration of muyocopronone A (**1**) was established as the (2′*R*,3′*R*,4′*S*,7*S*,10*R*,11*R*) stereoisomer.

Muyocopronone B (**2**) was also obtained as yellow amorphous solid, and its ESIMS was consistent with the molecular formula C_25_H_32_O_7_, thereby suggesting the addition of two hydrogen atoms relative to **1**. A comparison of the ^13^C NMR and DEPT135 data of compound **2** with that of **1** indicated that two methylene groups (i.e., at δ_C_ 25.0 (C-13) and 41.0 (C-14)) were present in **2** instead of the vicinal-coupled olefinic protons observed in the case of **1** (Table 1). Therefore, the structure of **2** was suggested to be the compound in which the double bond between C-10 and C-11 in **1** was hydrogenated. In addition, the presence of a 6-methyl-2-oxocyclohex-1-yl substituent was confirmed by the DQF-COSY correlations of H-10/H-11/H_3_-16 and H_2_-13/H_2_-14, and the HMBC correlations of H-10/C-15, H-12β/C-10, H-13β/C-15, H-14α/C-10, C-15, and H_3_-16/C-10, C-11, C-12. The absolute configuration of the side chain of **2** was also suggested to be the (2′*R*,3′*R*,4′*S*) configuration, which was confirmed using the modified Mosher’s method, whereby similar results were obtained as for compound **1** (Scheme S1, Supporting Information File 1). Furthermore, the calculated spectrum for the side chain-simplified model of the (7*S*,10*R*,11*R*)-stereoisomer was in good agreement with the experimental spectrum of **1** (Figure 4B and Figure S1, Supporting Information File 1). Therefore, the absolute configuration of **2** was also determined to be the (2′*R*,3′*R*,4′*S*,7*S*,10*R*,11*R*) stereoisomer.

Finally, since a number of azaphilones have been reported to exhibit antimicrobial activity [5,23], muyocopronones A (**1**) and B (**2**) were screened for their antibacterial activity against five strains of Gram-positive bacteria, namely *Staphylococcus aureus* (PAGU 273^T^), methicillin-resistant *S. aureus* (PAGU 841, MRSA), *S. epidermidis* (PAGU 283^T^), *Enterococcus faecalis* (PAGU 102^T^), and vancomycin-resistant *E. faecalis* (PAGU 100). Although muyocopronone B (**2**) exhibited an antibacterial activity against both antibiotic-resistant and antibiotic-susceptible strains, with minimum inhibitory concentration (MIC) values of ≈128 μg/mL (Table S1, Supporting Information File 1), the antibacterial activity of **2** was extremely weak compared to those of previously reported antibacterial azaphilones.

Azaphilones containing 6-membered rings constitute less than 10% of the hundreds of azaphilones isolated to date [5], and to the best of our knowledge, only eight compounds with a 2,4-dimethyl-3-hydroxyhexanoate moiety have been previously reported. More specifically, three thiodiketopiperazines, namely SCH 64874, 64875, and 64877, were isolated from the fermentation broth of an unidentified fungus [20,26], while three azaphilone-related polyketides, namely dothideomycetones A, B, and dothideomycetide A, were isolated from the CR17 fungal strain of the Dothideomycetes class (GeneBank accession number JQ867364) [27]. Furthermore, two sesquiterpenoids produced by *Pithomyces chartarum* (Dothideomycetes) were reported as intercellular adhesion molecule-1 (ICAM1) expression inhibitors in a patent literature [28]. Therefore, the presence of the 2,4-dimethyl-3-hydroxyhexanoate moiety may be a potential chemical marker for Dothideomycetes, although this is difficult to conclude due to the fact that few such compounds have been previously reported.

## Conclusion

In summary, we successfully performed the isolation and structural determination of muyocopronones A (**1**) and B (**2**) from the cultures of an endophytic fungus *Muyocopron laterale* ECN279. Muyocopronones A (**1**) and B (**2**) are both azaphilones containing 6-membered rings at the C-3 position of the azaphilone core and have a 2,4-dimethyl-3-hydroxyhexanoate moiety. Muyocopronone B (**2**) exhibited very weak antibacterial activity against both antibiotic-resistant and antibiotic-susceptible strains of Gram-positive bacteria.

## Experimental

### General experimental procedures

Optical rotation values were recorded on a JASCO P-2200 polarimeter, while the UV spectra were obtained using a Hitachi U-2900 spectrophotometer. The ECD spectra were acquired on a JASCO J-820 spectropolarimeter and the IR spectra were recorded on a Shimadzu FTIR-8400S spectrophotometer. NMR spectra were acquired on a JEOL JNM-ECZ 400S spectrometer with tetramethylsilane as the internal standard. ESIMS data were obtained using an Agilent 6230B TOF LC/MS system equipped with an electrospray ion source (Agilent Technologies, CA, USA) and an Agilent 1260 infinity II LC (Agilent Technologies). DNA sequencing was performed using an Applied Biosystems 3130 genetic analyzer. Silica gel AP-300 (Toyota Kako) was employed for column chromatography (CC). Silica gel 60 F_254_ and RP-18 F_254S_ (both Merck) were used for TLC.

### Fungal material and identification

*Muyocopron laterale* ECN279 was isolated from a healthy leaf of *Canavalia lineata* (Thunb.) DC collected at Tanegashima, Kagoshima, Japan. Strain isolation was performed using a previously described method [13]. Based on the DNA sequencing of the rDNA ITS and the D1/D2 domain of the 26S rDNA, the isolate was identified as *Muyocopron laterale*. The sequence data for *Muyocopron laterale* ECN279 have been deposited at the DNA Data Bank of Japan (DDBJ) under access numbers LC541569 (ITS) and LC541568 (26S rRNA) (Figures S2 and S3, Supporting Information File 1). The strain was deposited at the Department of Microbiology, School of Pharmacy, Aichi Gakuin University (ECN-279).

### Fermentation, extraction, and isolation

The fungus *Muyocopron laterale* ECN279 was inoculated onto 50 malt extract agar plates. After incubation at 27 °C for 30 d, the fermented materials were extracted with MeOH (1 L × 3 times, each 48 h) at room temperature (ca. 25 °C), and the solution was evaporated in vacuo to afford the MeOH extract (9.9 g). The MeOH extract was then partitioned twice with equal amounts of ethyl acetate and water, and the ethyl acetate solution was concentrated under vacuum to yield the ethyl acetate soluble fraction (6.6 g). This ethyl acetate fraction was then separated by silica gel CC using CHCl_3_/acetone (stepwise gradient, 1:0, 25:1, 10:1, and 0:1 v/v) as the eluent. The fractions were combined according to TLC analysis to yield five fractions. Fraction 2 was recrystallized in MeOH to yield **3** (96.9 mg), while fraction 3 was subjected to silica gel CC with *n*-hexane/ethyl acetate (stepwise gradient, 3:1, 2:1, and 1:1 v/v) to obtain compound **4** (37.3 mg). Fraction 5 was also subjected to silica gel CC with *n*-hexane/ethyl acetate (stepwise gradient, 4:1, 3:1, 2:1, and 1:1 v/v) to obtain compounds **2** (263.2 mg) and **1** (241.6 mg) eluted at solvent mixture ratio of 2:1 and 1:1, respectively.

### Muyocopronone A (**1**)

Yellow amorphous solid; [α]_D_^22^ +41 (*c* 0.1, MeOH); UV (MeOH) λ_max_ (log ε) 221 (4.34), 329 (4.25) nm; ECD (0.02 mg/mL, MeOH) λ_ext_ (Δε) 219 (−2.48), 236 (−4.29), 255 (+1.10), 276 (−1.37), 326 (+2.05), 358 (+2.25) nm; IR (KBr) ν_max_: 3468, 2963, 2934, 2876, 1717, 1674, 1634, 1553, 1456, 1387, 1335, 1314, 1231, 1177, 1121, 1086, 972, 914, 878 cm^−1^; ^1^H and ^13^C NMR data, see Table 1; HRESIMS (*m/z*): [M + H]^+^ calcd for C_25_H_31_O_7_, 443.2064; found, 443.2073.

### Muyocopronone B (**2**)

Yellow amorphous solid; [α]_D_^22^ +73 (*c* 0.1, MeOH); UV (MeOH) λ_max_ (log ε) 218 (4.14), 331 (4.28) nm; ECD (0.02 mg/mL, MeOH) λ_ext_ (Δε) 219 (−2.80), 253 (+2.10), 275 (−1.99), 351 (+4.19) nm; IR (KBr) ν_max_: 3480, 2963, 2936, 2876, 1717, 1669, 1634, 1551, 1456, 1373, 1332, 1267, 1231, 1184, 1123, 1088, 970, 914, 878, 735 cm^−1^; ^1^H and ^13^C NMR data, see Table 1; HRESIMS (*m/z*): [M + H]^+^ calcd for C_25_H_33_O_7_, 445.2221; found, 445.2228.

### Preparation of the MTPA diesters **1a** and **1b**

To a solution of **1** (12.4 mg, 0.028 mmol), DMAP (12.4 mg, 0.10 mmol), and triethylamine (15 μL, 0.11 mmol) in CH_2_Cl_2_ (1.2 mL) was added (*R*)-MTPACl (14 μL, 0.075 mmol). After allowing to stand in the dark at 25 °C for 6 h, the resulting solution was directly subjected to silica gel CC with *n*-hexane/ethyl acetate (1:1) to obtain (*S*)-MTPA diester **1a** (10.5 mg). Similarly, a mixture of **1** (7.2 mg, 0.016 mmol), DMAP (7.2 mg, 0.059 mmol), and triethylamine (8.7 μL, 0.064 mmol) in CH_2_Cl_2_ (1.0 mL) was treated with (*S*)-MTPACl (8.0 μL, 0.043 mmol) in the same manner to obtain (*R*)-MTPA diester **1b** (3.5 mg).

### (*S*)-MTPA diester **1a**

Yellow gum; ^1^H NMR *δ_H_* 0.92 (d, *J* = 6.9 Hz, 3H, H-8′), 0.95 (t, *J* = 7.3 Hz, 3H, H-6′), 1.16 (d, *J* = 7.3 Hz, 3H, H-16), 1.21 (m, 1H, H_a_-5′), 1.25 (d, *J* = 7.3 Hz, 3H, H-7′), 1.45 (s, 3H, H-9), 1.49 (m, 1H, H_b_-5′), 1.87 (m, 1H, H-4′), 2.26 (ddd, *J* = 1.8, 6.7, 18.1 Hz, 1H, H-12), 2.66 (dddd, *J* = 2.5, 2.7, 6.0 18.1 Hz, 1H, H-12), 2.85 (qd, *J* = 6.7, 7.3 Hz, 1H, H-11), 3.08 (br dq, *J* = 7.2, 7.3 Hz, 1H, H-2′), 3.49 and 3.58 (6H, methoxy groups of MTPA), 5.28 (d, *J* = 1.4 Hz, 1H, H-5), 5.41 (dd, *J* = 4.8, 7.2 Hz, 1H, H-3′), 5.85 (dd, *J* = 2.7, 10.1 Hz, 1H, H-14), 6.16 (ddd, *J* = 2.5, 6.0, 10.1 Hz, 1H, H-13), 6.34 (s, 1H, H-4), 7.27 (1H, overlapped with the solvent signal, H-1), 7.31–7.44 (m, 10H, phenyl groups of MTPA).

### (*R*)-MTPA diester **1b**

Yellow gum; ^1^H NMR *δ_H_* 0.76 (d, *J* = 6.4 Hz, 3H, H-8′), 0.89 (t, *J* = 7.3 Hz, 3H, H-6′), 1.07 (m, 1H, H_a_-5′), 1.16 (d, *J* = 6.9 Hz, 3H, H-16), 1.28 (d, *J* = 7.3 Hz, 3H, H-7′), 1.36 (m, 1H, H_b_-5′), 1.49 (s, 3H, H-9), 1.81 (m, 1H, H-4′), 2.26 (ddd, *J* = 1.8, 6.4, 18.3 Hz, 1H, H-12), 2.68 (dddd, *J* = 2.7, 2.7, 8.2, 18.3 Hz, 1H, H-12), 2.85 (br dq, *J* = 6.9, 8.2 Hz, 1H, H-11), 3.08 (qd, *J* = 7.1, 7.3 Hz, 1H, H-2′), 3.59 and 3.62 (6H, methoxy groups of MTPAs), 5.28 (d, *J* = 0.9 Hz, 1H, H-5), 5.40 (dd, *J* = 5.0, 7.1 Hz, 1H, H-3′), 5.85 (dd, *J* = 2.7, 10.1 Hz, 1H, H-14), 6.16 (ddd, *J* = 2.7, 6.4, 10.1 Hz, 1H, H-13), 6.31 (s, 1H, H-4), 7.28 (d, *J* = 0.9 Hz, 1H, H-1), 7.33–7.62 (m, 10H, phenyl groups of MTPAs).

### Computational methods

Calculation of the ECD spectra were performed using CONFLEX 8, Gaussian 16, and SpecDis software as described previously [13–14]. Geometry optimizations were performed using DFT at the CAM-B3LYP/6-311+G(d,p) level of theory, while TDDFT calculations were conducted at the B3LYP/6-311+G(d,p) level of theory. The calculated spectra were displayed using a Gaussian band shape with 0.28 eV.

### Antibacterial assays

To evaluate the antibacterial activities of the isolated substances, five strains of Gram-positive bacteria were used, namely *Staphylococcus aureus* (PAGU 273^T^), methicillin-resistant *S. aureus* (PAGU 841, MRSA), *Staphylococcus epidermidis* (PAGU 283^T^), *Enterococcus faecalis* (PAGU 102^T^), and vancomycin-resistant *E. faecalis* (PAGU 100, VRE). Piperacillin, vancomycin, and amikacin were used as positive controls. The isolated substances and the antibiotics were serially diluted (over a concentration range of 1–1024 μg/mL) into the wells of a 96-well plate. Following addition of the bacterial inoculum, the microtiter plate was incubated at 37 °C for 18 h. The microorganisms were cultured in CAMHB (cation-adjusted Mueller-Hinton broth) medium at a concentration of 1 × 10^6^ CFU/mL. The recorded MIC was the lowest concentration at which no growth was observed.

## Supporting Information

File 1 Preparations of MTPA diesters **2a** and **2b**, phylograms of ECN-279 and related species, and copies of 1D and 2D NMR data for **1** and **2**.
