# Supplementary material for: Muyocopronones A and B: azaphilones from the endophytic fungus Muyocopron laterale
Source: Beilstein J Org Chem. 2020 Aug 28;16:2100–7. doi: 10.3762/bjoc.16.177 (PMC7476592; doi:10.3762/bjoc.16.177)
Supplement: File 1 — Preparations of MTPA diesters 2a and 2b, phylograms of ECN-279 and related species, and copies of 1D and 2D NMR data for 1 and 2. [file Beilstein_J_Org_Chem-16-2100-s001.pdf]

BEILSTEIN JOURNAL OF ORGANIC CHEMISTRY

Supporting Information

for

Muyocopronones A and B: azaphilones from the endophytic fungus *Muyocopron laterale*

Ken-ichi Nakashima, Junko Tomida, Tomoe Tsuboi, Yoshiaki Kawamura and Makoto Inoue

Beilstein J. Org. Chem. **2020,** *16,* 2100–2107. doi:10.3762/bjoc.16.177

Preparations of MTPA diesters 2a and 2b, phylograms of ECN-279 and related species, and copies of 1D and 2D NMR data for 1 and 2

License and Terms: This is a supporting information file under the terms of the Creative Commons Attribution License (https://creativecommons.org/licenses/by/4.0). Please note that the reuse, redistribution and reproduction in particular requires that the authors and source are credited.
The license is subject to the *Beilstein Journal of Organic Chemistry* terms and conditions: (https://www.beilstein-journals.org/bjoc)

Table of contents

Preparations of MTPA diesters 2a and 2b ··· S2

Scheme S1. (A) Synthesis of MTPA diesters **2a** and **2b**. (B) Δ*δ* values for (*S*)- and (*R*)-MTPA esters of **2**. ··· S3

Figure S1. Simplified models for calculations of ECD spectra of **2**. ··· S3

Figure S2. Phylogram obtained from ITS of rDNA sequences of ECN-279 and related species. ··· S4

Figure S3. Phylogram obtained from D1/D2 domain of 26SrRNA sequences of ECN-279 and related species. ··· S5

Figure S4. ^{1}H NMR spectrum (400 MHz, $CDCl_3$) of muyocopronone A (**1**). ··· S6

Figure S5. ^{13}C NMR and DEPT135 spectra (100 MHz, $CDCl_3$) of muyocopronone A (**1**). S7

Figure S6. DQF-COSY spectrum ($CDCl_3$) of muyocopronone A (**1**). ··· S8

Figure S7. HSQC spectrum ($CDCl_3$) of muyocopronone A (**1**). ··· S9

Figure S8. HMBC spectrum ($CDCl_3$) of muyocopronone A (**1**). ··· S10

Figure S9. ^{1}H NMR spectrum (400 MHz, $CDCl_3$) of muyocopronone B (**2**). ··· S11

Figure S10. ^{13}C NMR and DEPT135 spectra (100 MHz, $CDCl_3$) of muyocopronone B (**2**). S12

Figure S11. DQF-COSY spectrum ($CDCl_3$) of muyocopronone B (**2**). ··· S13

Figure S12. HSQC spectrum ($CDCl_3$) of muyocopronone B (**2**). ··· S14

Figure S13. HMBC spectrum ($CDCl_3$) of muyocopronone B (**2**). ··· S15

Figure S14. IR spectrum of muyocopronone A (**1**). ··· S16

Figure S15. IR spectrum of muyocopronone B (**2**). ··· S16

Table S1. Antibacterial activities of **1** and **2** ··· S17

Preparations of MTPA diesters 2a and 2b

To a solution of **2** (4.0 mg, 0.009 mmol) with DMAP (4.0 mg, 0.033 mmol) and triethylamine (4.9 µL, 0.035 mmol) in CH_2Cl_2 (1.0 mL) was added (*R*)-MTPACl (4.8 µL, 0.026 mmol). After standing still at dark place (25 ºC) for 7 h, the resulting solution was directly subjected to silica gel CC with *n*-hexane/ethyl acetate (1:1) to obtain the (*S*)-MTPA diester **2a** (2.8 mg). Similarly, the mixture of **2** (4.0 mg, 0.009 mmol), DMAP (4.0 mg, 0.033 mmol), and triethylamine (4.9 µL, 0.035 mmol) in CH_2Cl_2 (1.0 mL) was treated with (*S*)-MTPACl (4.8 µL, 0.026 mmol) by the same manner as above to obtain (*R*)-MTPA diester **2b** (1.7 mg).

*(S)-MTPA diester **2a**:* yellow gum; ^{1}H NMR δ_H 0.92 (3H, d, *J* = 6.4 Hz, H-8′), 0.96 (3H, t, *J* = 7.3 Hz, H-6′), 1.09 (3H, d, *J* = 6.9 Hz, H-16), 1.21 (1H, m, H_a-5′), 1.23 (3H, d, *J* = 7.3 Hz, H-7′), 1.26 (1H, m), 1.45 (3H, s, H-9), 1.51 (1H, m, H_b-5′), 1.76–1.91 (4H, m), 2.29–2.41 (2H, m), 2.78 (1H, m, H-11), 3.09 (1H, br dq, *J* = 6.9, 7.3 Hz, H-2′), 3.50 (6H, methoxy groups of MTPA), 5.35 (1H, d, *J* = 0.9 Hz, H-5), 5.41 (1H, dd, *J* = 5.0, 6.9 Hz, H-3′), 6.16 (1H, s, H-4), 7.34–7.62 (11H, m, phenyl groups of MTPA and H-1).

*(R)-MTPA diester **2b**:* yellow gum; ^{1}H NMR δ_H 0.76 (3H, d, *J* = 6.9 Hz, H-8′), 0.89 (3H, t, *J* = 7.3 Hz, H-6′), 1.06 (3H, d, *J* = 6.9 Hz, H-16), 1.07–1.12 (1H, overlapped signal, H_a-5′), 1.27 (3H, d, *J* = 7.3 Hz, H-7′), 1.36 (1H, m, H_b-5′), 1.50 (3H, s, H-9), 1.76–1.92 (4H, m), 2.25–2.44 (2H, m), 2.77 (1H, m, H-11), 3.09 (1H, qd, *J* = 6.9, 7.3 Hz, H-2′), 3.54 and 3.59 (6H, methoxy groups of MTPAs), 5.36 (1H, d, *J* = 0.9 Hz, H-5), 5.41 (1H, dd, *J* = 5.0, 6.9 Hz, H-3′), 6.16 (1H, s, H-4), 7.35–7.64 (11H, m, phenyl groups of MTPA and H-1).

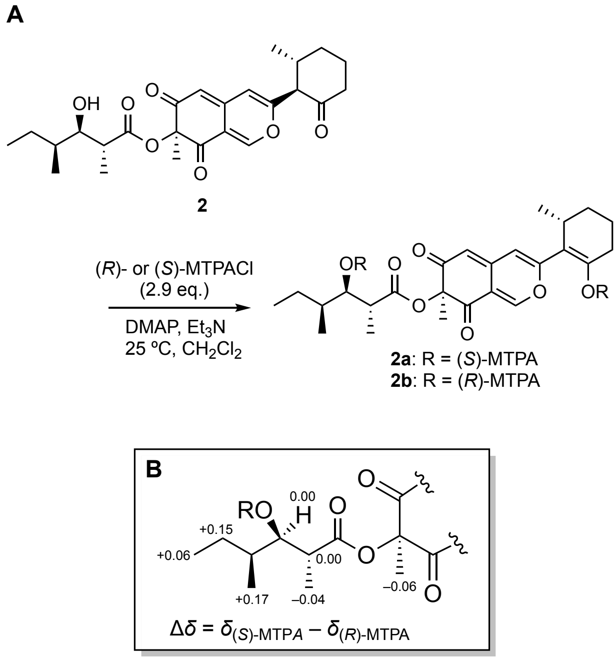

Scheme S1: (A) Synthesis of MTPA diesters **2a** and **2b**. (B) Δ*δ* values for (*S*)- and (*R*)-MTPA esters of **2**. Δ*δ* values of C-4′ and C-5′b could not be calculated due to overlap with other signals.

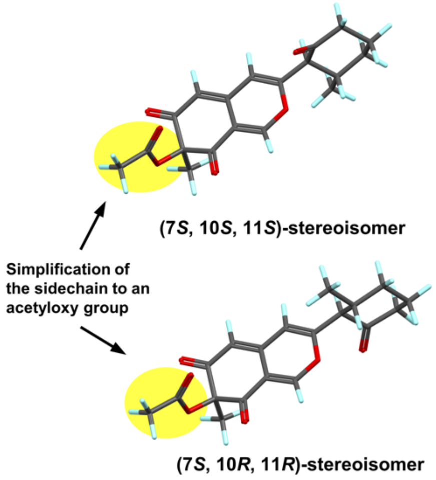

Figure S1: Simplified models for calculations of ECD spectra of **2**.

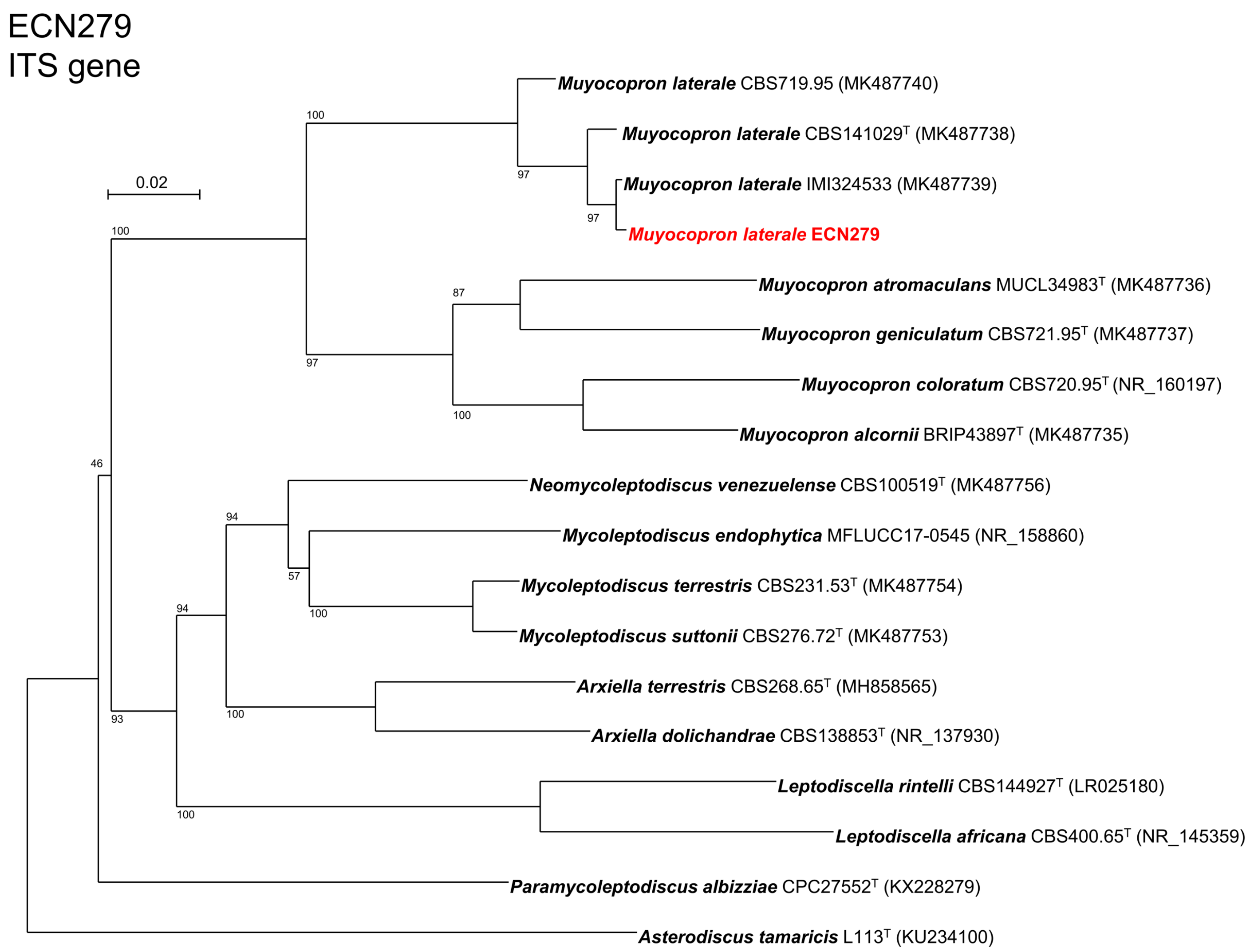

Figure S2: Phylogram obtained from ITS of rDNA sequences of ECN-279 and related species.

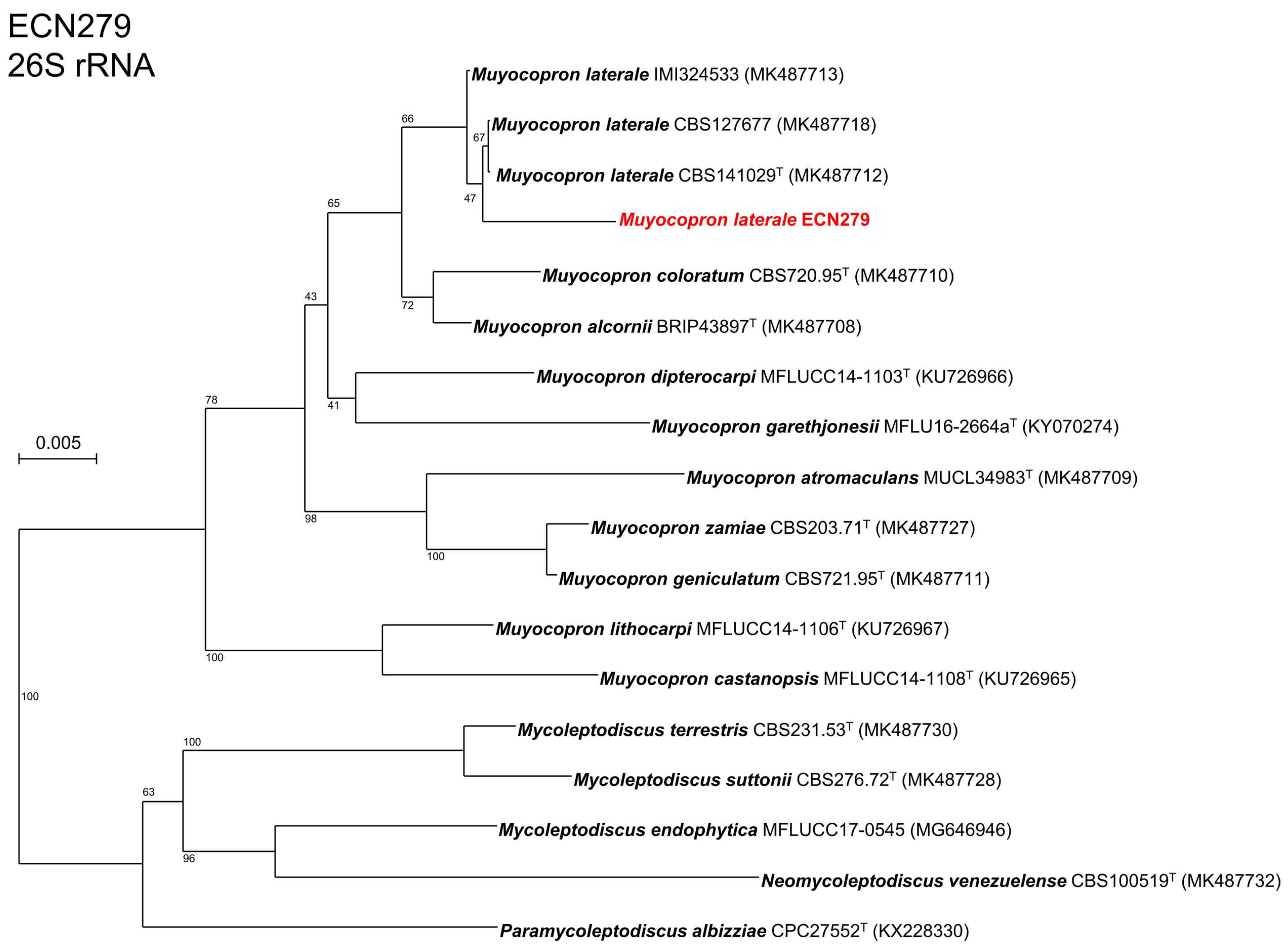

Figure S3: Phylogram obtained from D1/D2 domain of 26SrRNA sequences of ECN-279 and related species.

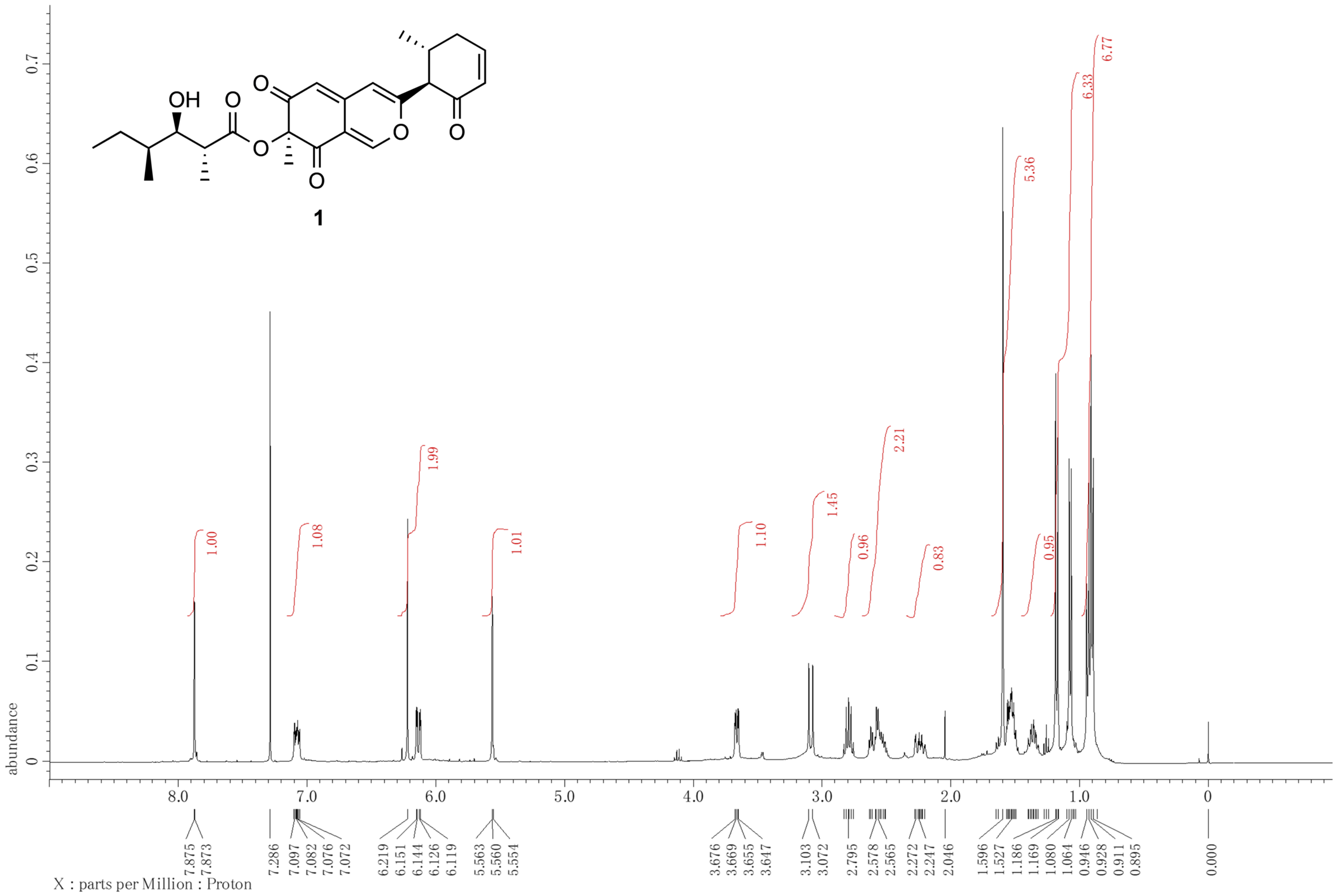

Figure S4: ^{1}H NMR spectrum (400 MHz, $CDCl_3$) of muyocopronone A (**1**).

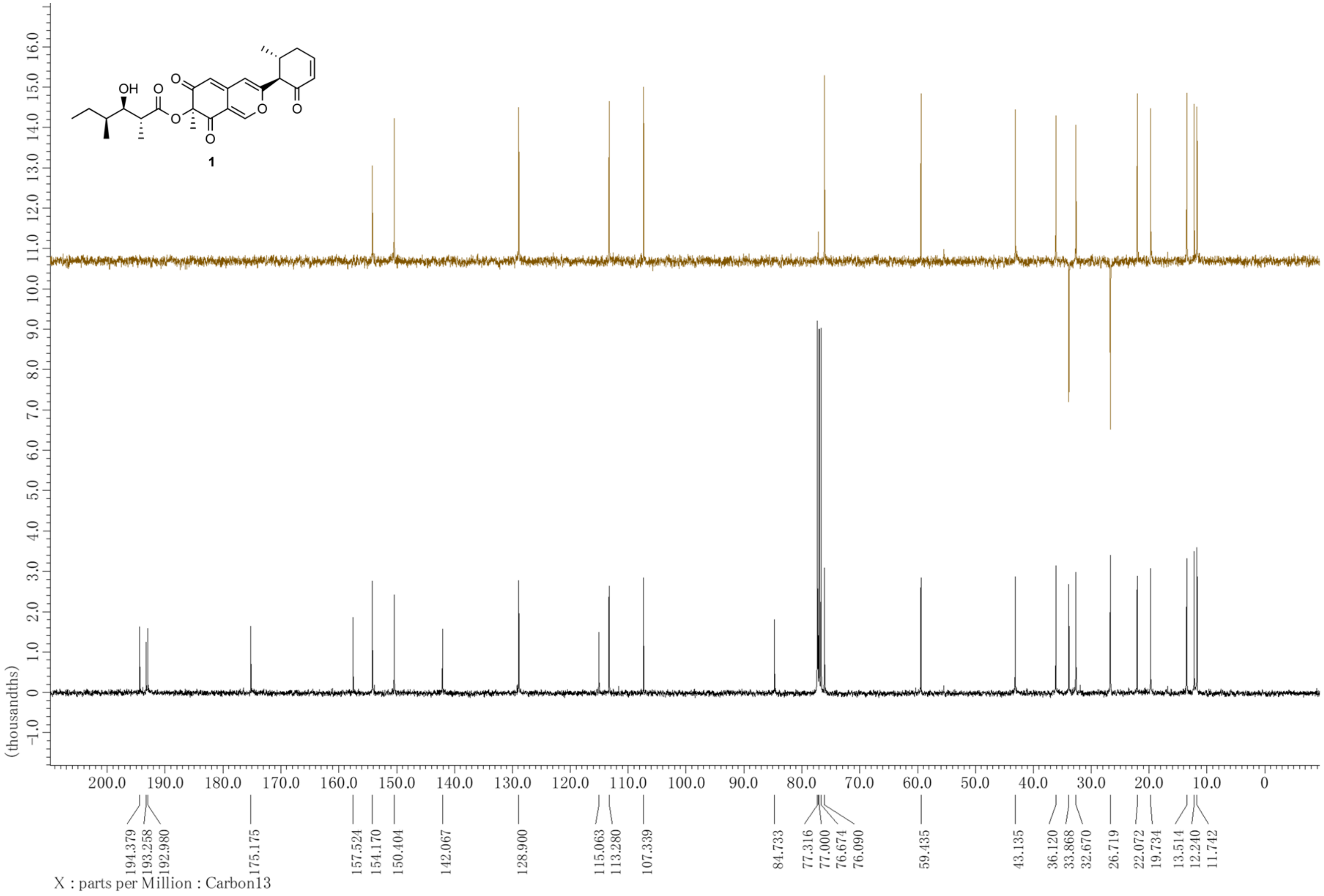

Figure S5: ^{13}C NMR and DEPT135 spectra (100 MHz, $CDCl_3$) of muyocopronone A (**1**).

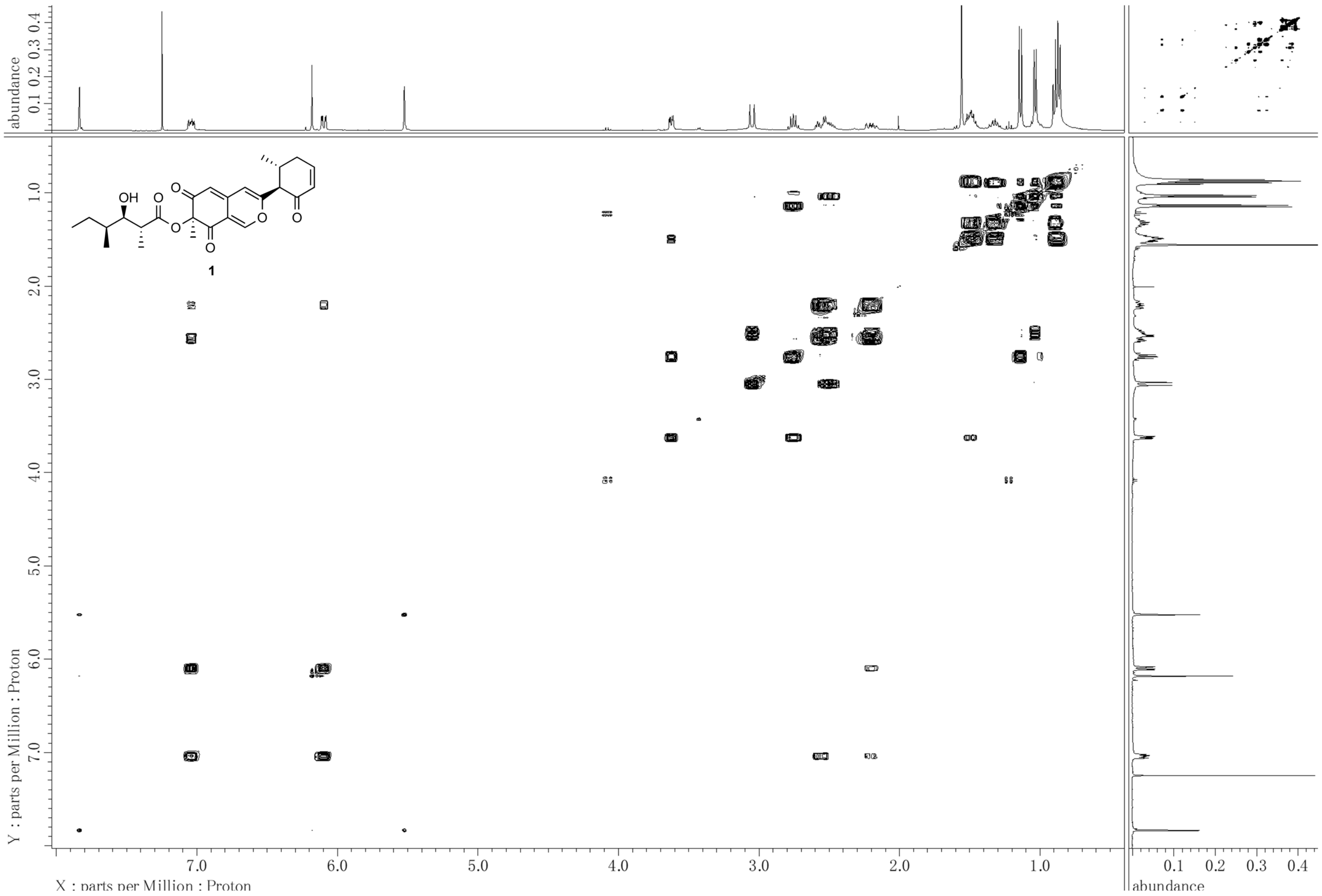

Figure S6: DQF-COSY spectrum ($CDCl_3$) of muyocopronone A (**1**).

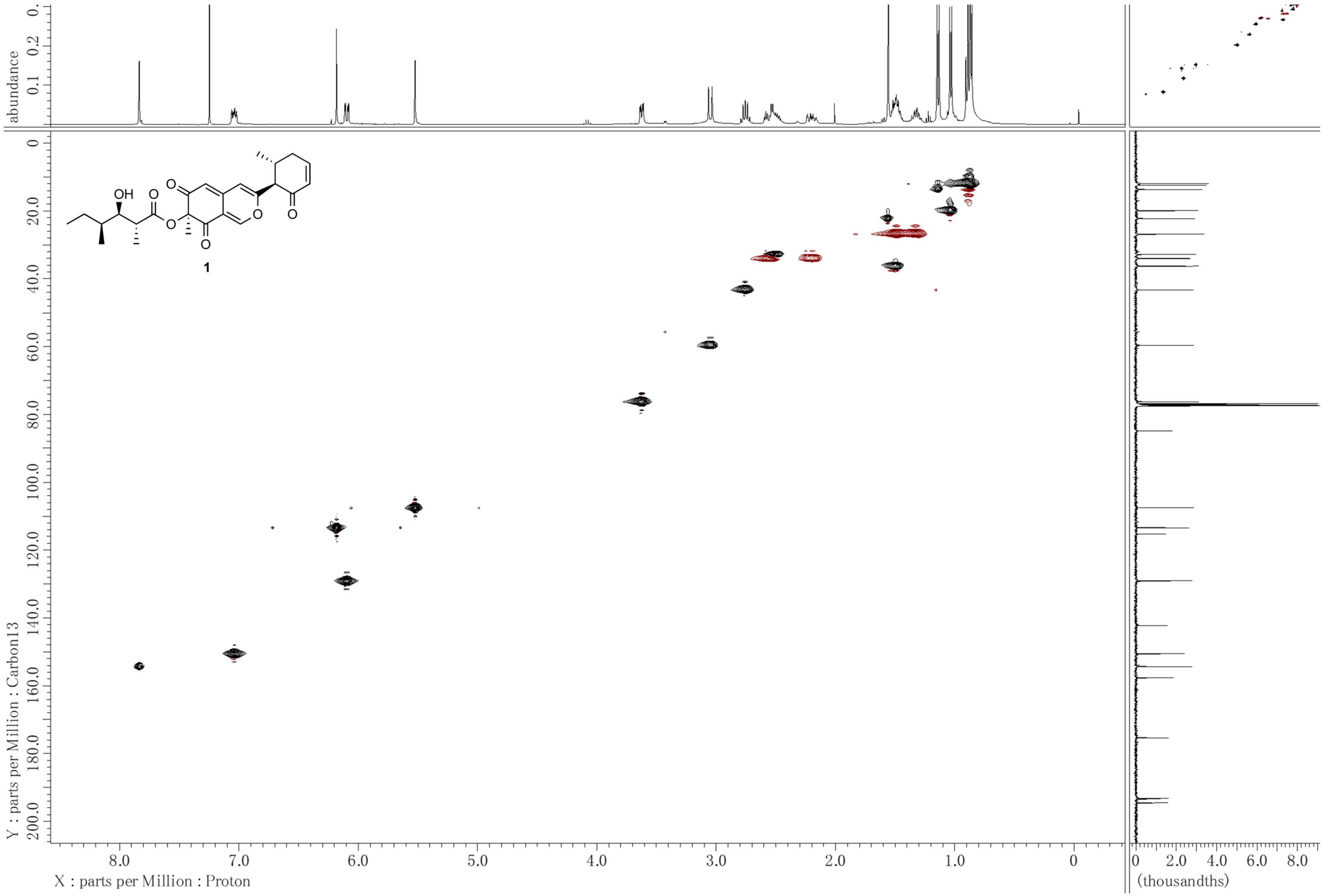

Figure S7: HSQC spectrum ($CDCl_3$) of muyocopronone A (**1**).

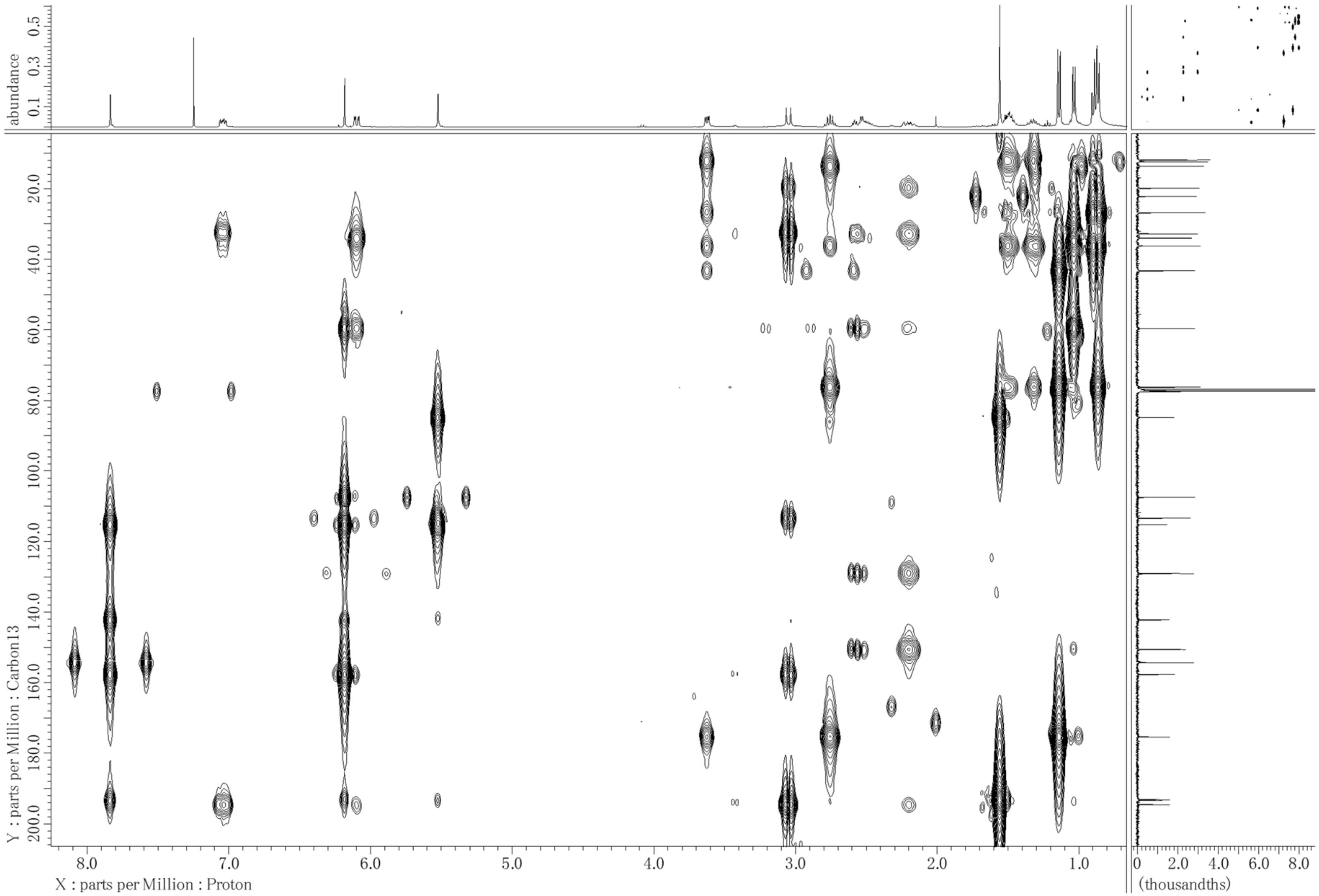

Figure S8: HMBC spectrum ($CDCl_3$) of muyocopronone A (**1**).

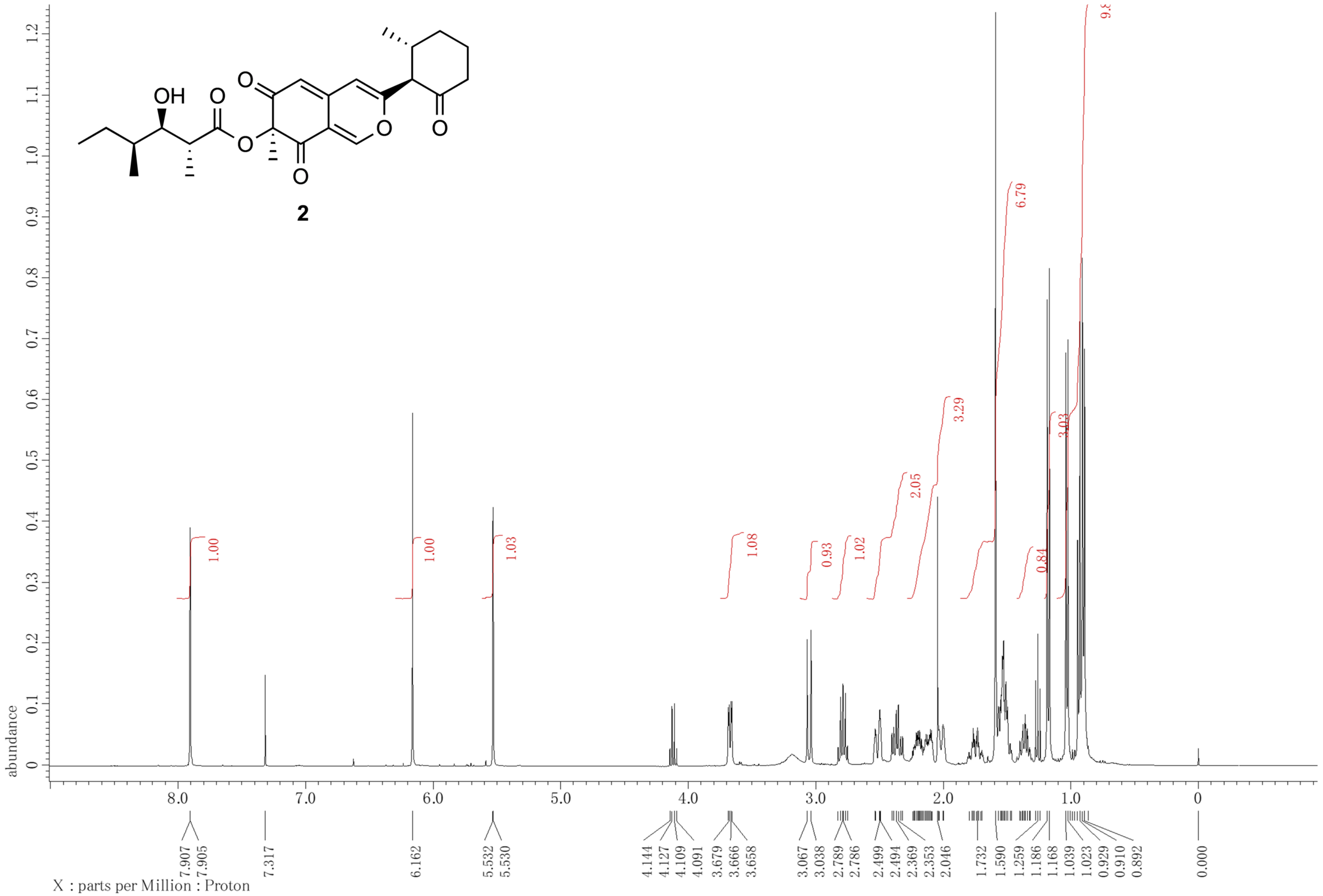

Figure S9: ^{1}H NMR spectrum (400 MHz, $CDCl_3$) of muyocopronone B (**2**).

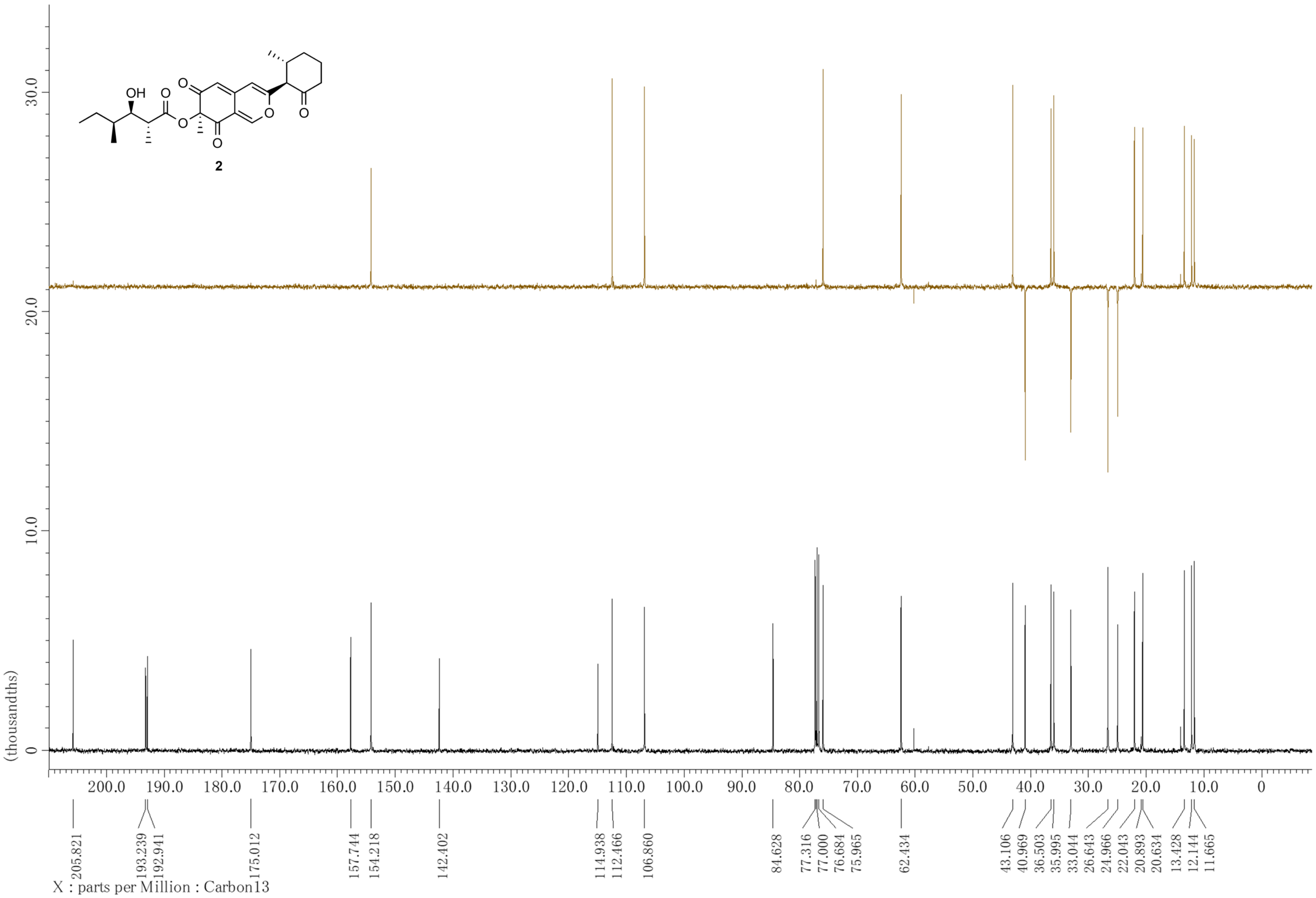

Figure S10: ^{13}C NMR and DEPT135 spectra (100 MHz, $CDCl_3$) of muyocopronone B (**2**).

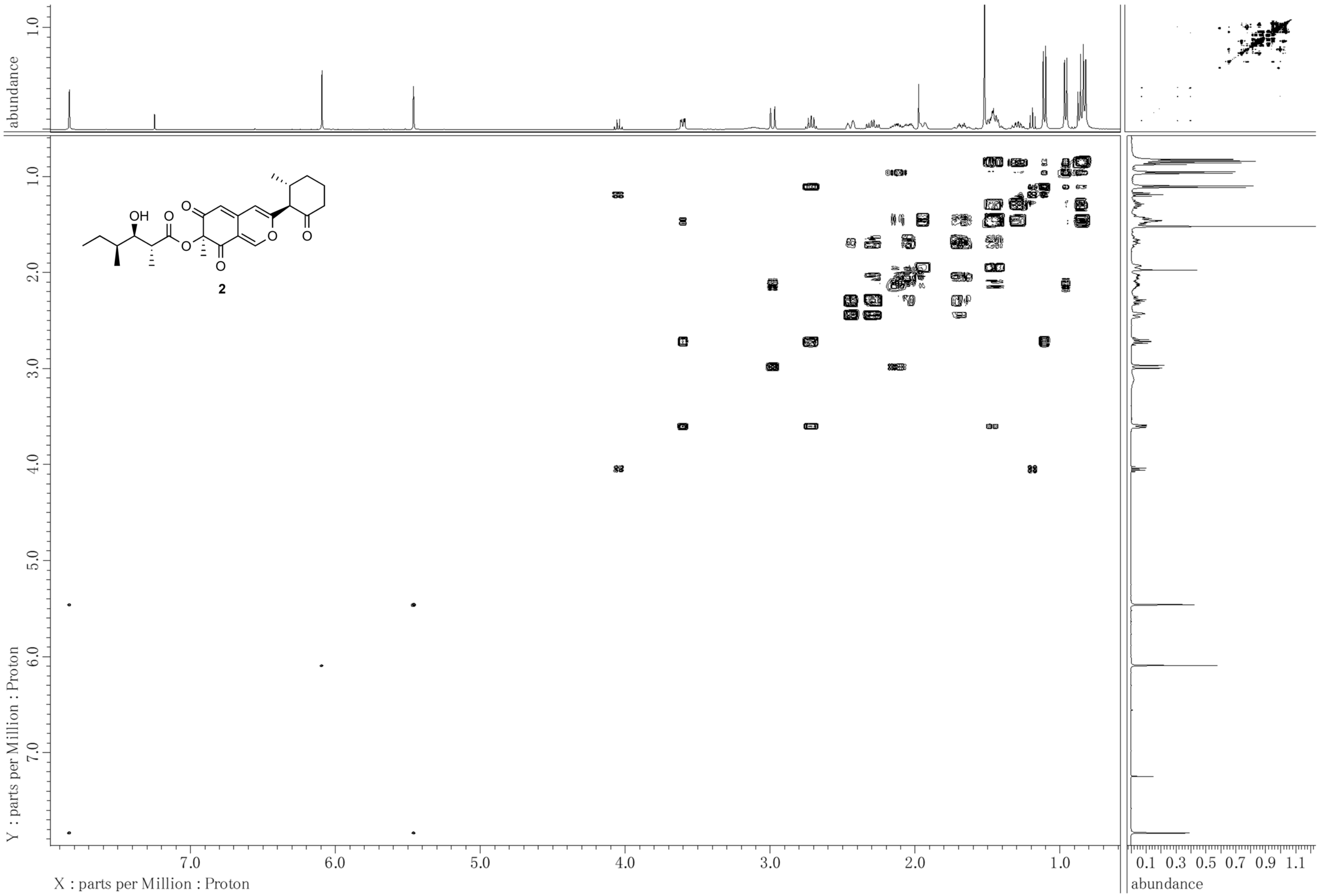

Figure S11: DQF-COSY spectrum ($CDCl_3$) of muyocopronone B (**2**).

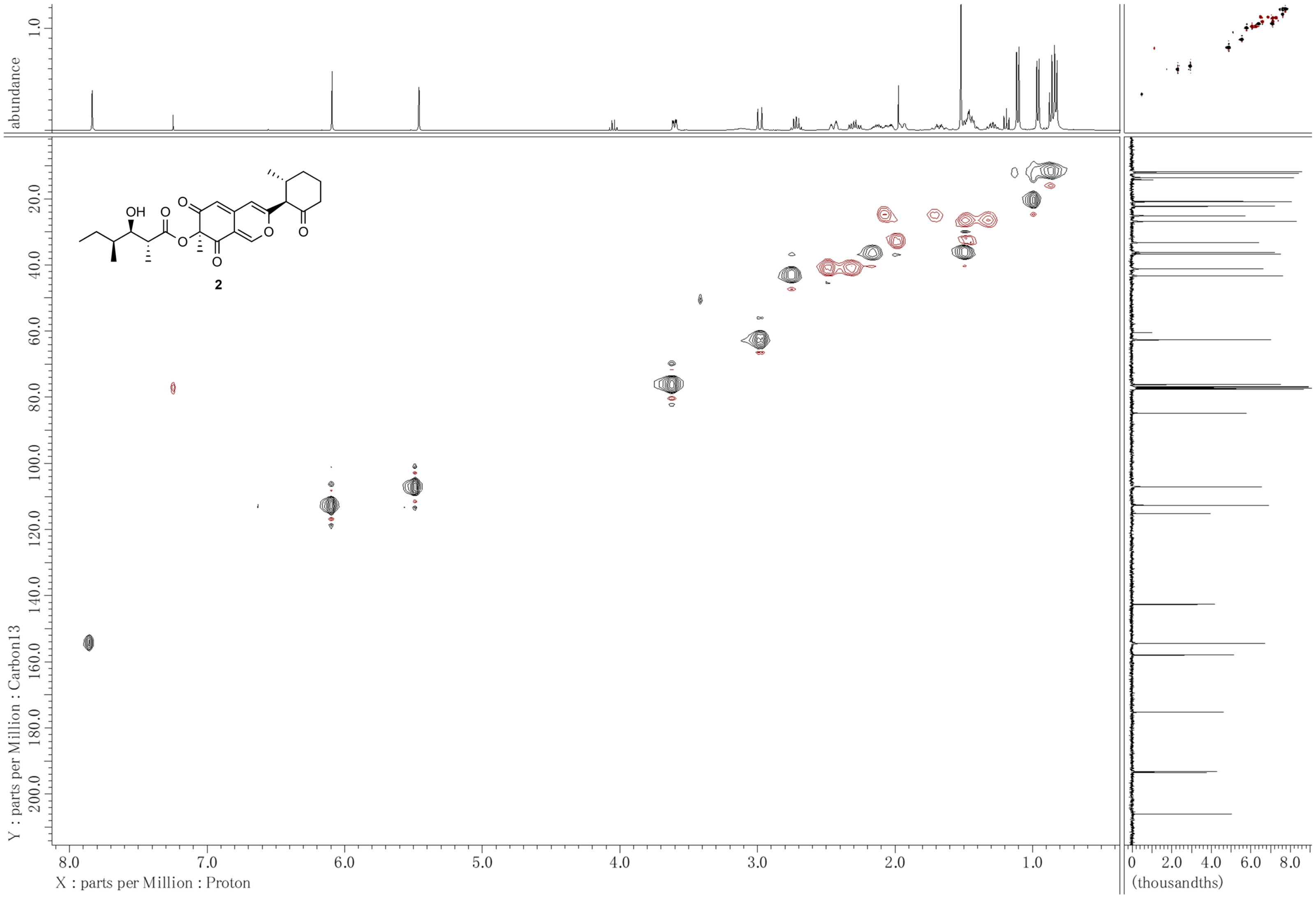

Figure S12: HSQC spectrum ($CDCl_3$) of muyocopronone B (**2**).

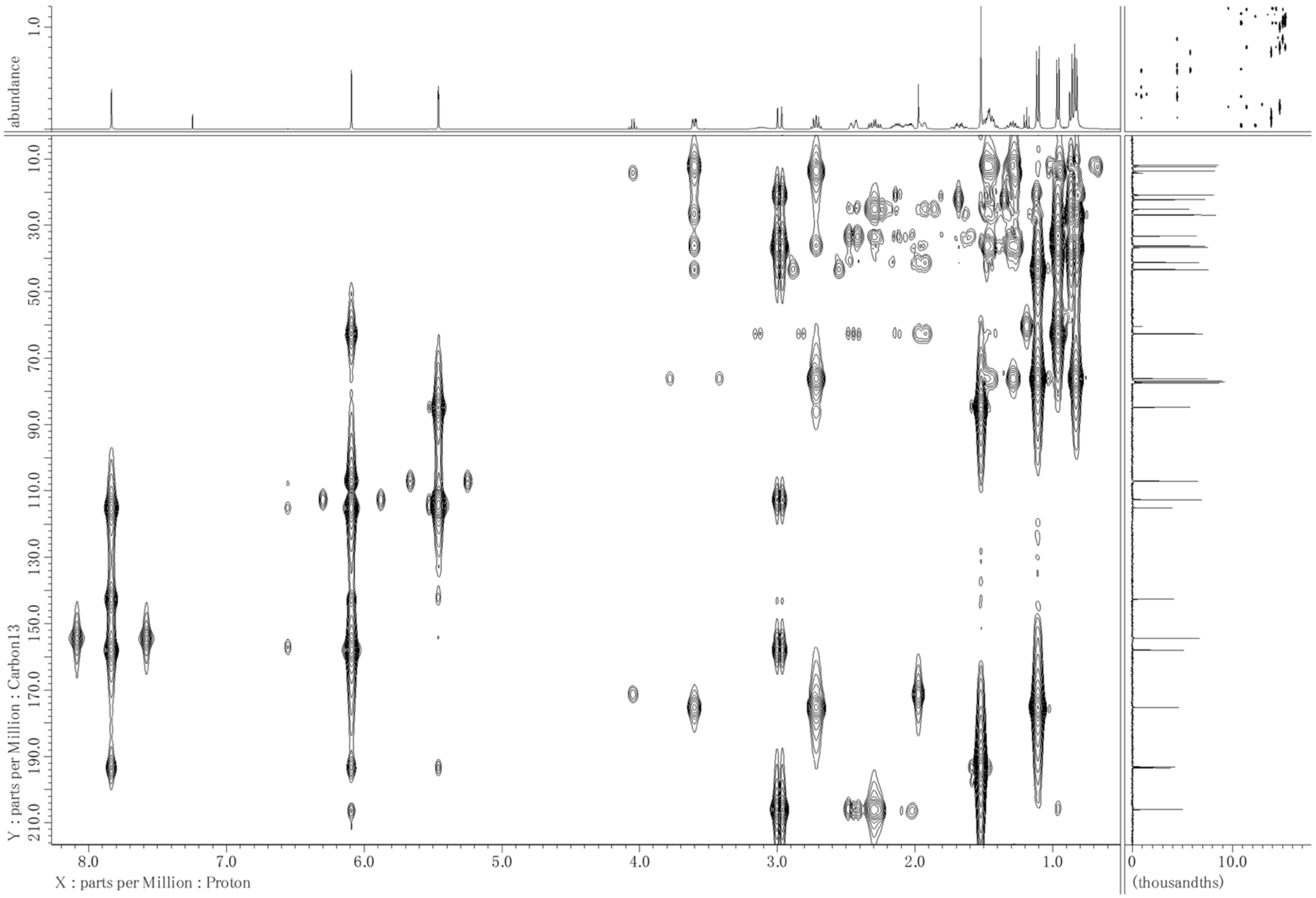

Figure S13: HMBC spectrum ($CDCl_3$) of muyocopronone B (**2**).

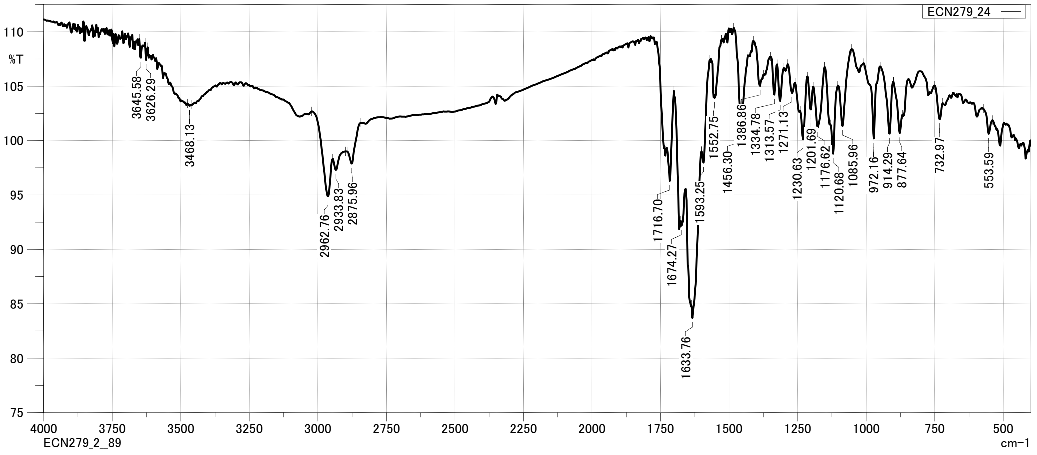

Figure S14: IR spectrum of muyocopronone A (**1**).

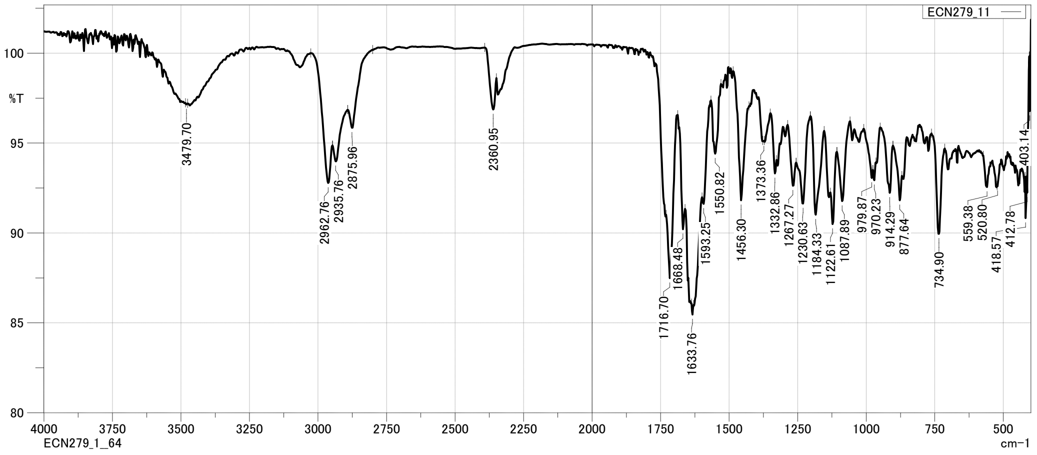

Figure S15: IR spectrum of muyocopronone B (**2**).

Table S1: Anti-bacterial activities of **1** and **2**

bacteria	Antibiotic resistant (–)/susceptible (+)	MIC (μg/mL) **1**	**2**	piperacillin	vancomycin	amikacin
Staphylococcus aureus PAGU 273[T]	methicillin (+)	1024	128	<1	<1	8
Staphylococcus aureus PAGU 841	methicillin (–)	>1024	128	512	1	4
Enterococcus faecalis PAG102[T]	vancomycin (+)	1024	128	<1	16	256
Enterococcus faecalis PAG100	vancomycin (–)	1024	128	2	<1	256
Staphylococcus epidermidis PAGU 283[T]		>1024	128	<1	<1	<1
